# Could the clinical interpretability of subgroups detected using clustering methods be improved by using a novel two-stage approach?

**DOI:** 10.1186/s12998-015-0064-9

**Published:** 2015-07-02

**Authors:** Peter Kent, Mette Jensen Stochkendahl, Henrik Wulff Christensen, Alice Kongsted

**Affiliations:** Department of Sports Science and Clinical Biomechanics, University of Southern Denmark, Campusvej 55, Odense, M 5230 Denmark; Nordic Institute of Chiropractic and Clinical Biomechanics, Odense, Denmark

**Keywords:** Stratified health care, Clustering, Latent class analysis, Subgroups, Chest pain, Low back pain

## Abstract

**Background:**

Recognition of homogeneous subgroups of patients can usefully improve prediction of their outcomes and the targeting of treatment. There are a number of research approaches that have been used to recognise homogeneity in such subgroups and to test their implications. One approach is to use statistical clustering techniques, such as Cluster Analysis or Latent Class Analysis, to detect latent relationships between patient characteristics.

Influential patient characteristics can come from diverse domains of health, such as pain, activity limitation, physical impairment, social role participation, psychological factors, biomarkers and imaging. However, such ‘whole person’ research may result in data-driven subgroups that are complex, difficult to interpret and challenging to recognise clinically.

This paper describes a novel approach to applying statistical clustering techniques that may improve the clinical interpretability of derived subgroups and reduce sample size requirements.

**Methods:**

This approach involves clustering in two sequential stages. The first stage involves clustering within health domains and therefore requires creating as many clustering models as there are health domains in the available data. This first stage produces scoring patterns within each domain. The second stage involves clustering using the scoring patterns from each health domain (from the first stage) to identify subgroups across all domains. We illustrate this using chest pain data from the baseline presentation of 580 patients.

**Results:**

The new two-stage clustering resulted in two subgroups that approximated the classic textbook descriptions of musculoskeletal chest pain and atypical angina chest pain. The traditional single-stage clustering resulted in five clusters that were also clinically recognisable but displayed less distinct differences.

**Conclusions:**

In this paper, a new approach to using clustering techniques to identify clinically useful subgroups of patients is suggested. Research designs, statistical methods and outcome metrics suitable for performing that testing are also described. This approach has potential benefits but requires broad testing, in multiple patient samples, to determine its clinical value. The usefulness of the approach is likely to be context-specific, depending on the characteristics of the available data and the research question being asked of it.

## Background

There is increasing interest in stratified health care that targets treatments to individuals or homogenous subgroups of patients. The potential benefits of stratified health care are better treatment effects and reduced harm through a more precise matching of therapy to individual patients, and improved health system efficiency through more appropriate resource allocation [1]. This is especially the case in health conditions with high diagnostic and therapeutic uncertainty, where randomised controlled trials of a ‘one size fits all’ treatment approach have yielded disappointing effect sizes [2].

It is usual for patients to vary in their outcomes, due to prognostic diversity and differences in their treatment response, even when their condition may appear to be similar at the initial consultation. Using knowledge of the predictability of that diversity, stratified health care attempts to use baseline information about likely prognosis and likely treatment response to assist in the tailoring of treatment decisions [1]. Under that model, clinical decisions are based on *predictions* of likely outcomes. In contrast, there are other models of care that use the patient’s *actual* response to treatment and initial outcomes to tailor subsequent treatment decisions, such as stepped care and adaptive treatment [3, 4].

Stratified health care requires prognostic models with sufficient predictive accuracy to be clinically useful. Such predictive accuracy either comes from an in-depth, although often elusive, understanding of what determines the variability in the outcomes of individual patients or more commonly, from recognising a sufficient amount of homogeneity in subgroups of patients that can usefully improve prediction of their outcomes [5].

There are a number of approaches that have been used to recognise homogeneity in subgroups of patients and to test its implications. The classic data-driven approach is to work backwards from an outcome in longitudinal data, such as people classified as responders or non-responders to a treatment regimen. As these techniques use the outcome to determine the derived subgroups, statisticians refer to these approaches as ‘supervised’ techniques [6, 7]. Such supervised statistical techniques include: regression analysis, discriminant function analysis, recursive partitioning analysis, and classification and regression trees. An example of a study using regression analysis to create a clinical prognostic tool is that performed by Schellingerhout et al. 2010 [8], who created a score chart to estimate the probability of non-recovery at 6-month follow-up in patients with non-specific neck pain. The other main approach used in data-driven subgrouping is called ‘unsupervised’ [6] [7] because instead of working backwards from an outcome, these statistical techniques look for latent relationships between characteristics in cross-sectional data, usually baseline data.

The main limitation of supervised techniques is that subgroup formation is usually based on the prediction of one single outcome that may not be a comprehensive measure of the behaviour of the condition (for example, predictors of return-to-work may not be the same as predictors of recovery from pain). In contrast, unsupervised subgroup formation is based on baseline data only, and such subgroups are not dependent on one outcome or the efficacy of current treatments. Therefore, an advantage of this approach is that subgroups detected in this way can later be studied against a range of treatments and outcomes. The major disadvantage of this method is that because subgroups are not modelled using a clinical outcome, subgroups derived using unsupervised techniques may have no clinical relevance [9]. Therefore, such subgroups need to be rigorously tested for predictive validity. Two examples of studies that used unsupervised clustering techniques to derive subgroups from baseline psychological data and then investigate their predictive validity in longitudinal data are those conducted by Beneciuk et al. 2012 [10] and Westman et al. 2011 [11].

There is an increasing recognition that achievement of high predictive accuracy is likely to require prognostic models derived from patient characteristics that cover all influential domains of health [12]. For example, in musculoskeletal conditions, there is compelling evidence that psychological and social factors play important prognostic roles [13, 14]. Increasingly therefore, prognostic research includes factors from such diverse domains as pain, activity limitation, physical impairment, work and social role participation, psychological factors, and biomedical testing (biomarkers and imaging).

However, such ‘whole person’ prognostic research may result in data-driven subgroups that are complex, difficult to interpret and challenging to recognise clinically. It would be useful if there were methodological approaches that harnessed the explanatory potential of that complexity while also facilitating clinical interpretability. An additional consideration is that modelling larger numbers of potentially predictive factors from multiple health domains requires larger patient sample sizes to avoid ‘overfitting’. Overfitting is present when an analysis excessively fits the available data and therefore has limited generalisability outside the available sample [15]. So, it would also be useful if there were methodological approaches that harnessed the explanatory potential of many predictive variables while also minimising the need for very large patient samples.

The main aim of this paper was to describe a novel approach to using statistical clustering techniques to identify subgroups of patients, an approach that theoretically may improve clinical interpretability and reduce sample size requirements. We used Latent Class Analysis [16] as an exemplar statistical clustering technique and non-specific low back pain as an exemplar health condition when explaining the conceptual approach. We chose low back pain because the potential usefulness of subgrouping in this condition is well recognised [17–19]. Subsequently, we used real chest pain data to illustrate a worked example, and chose these chest pain data because we had a suspicion that these data were likely to contain two latent subgroups. However, the approach may have potential application to other health conditions as well. The paper also suggests a framework for testing whether such subgroups are clinically important. The description of this novel approach is suggested as an option for researchers who use clustering techniques on complex clinical data and who have the intention of interpreting results in ways that are clinically meaningful.

## Methods

### Traditional approach to using statistical clustering techniques (low back pain example)

**Fig. 1:**
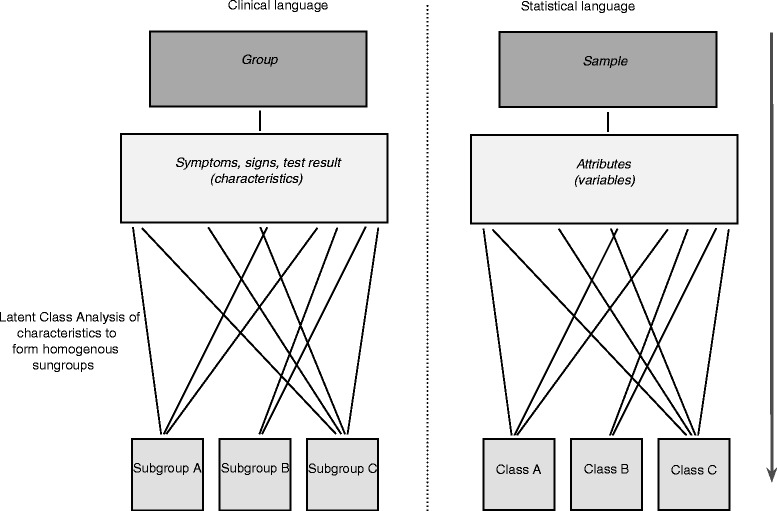
Traditional approach to using statistical clustering techniques and an illustration of different labels for the same statistical concepts

The traditional approach to using statistical clustering techniques to identify subgroups of patients is to enter selected clinical characteristics into a clustering model and then determine post-hoc in what ways the derived subgroups differ from each other. This general approach is illustrated in Fig. 1, which also shows how the same concept or attribute is named differently in clinical language and statistical language. To aid interpretability by clinically-orientated readers, this paper favours clinical language wherever possible.

For example, in a group of people with non-specific low back pain, we might select measures of various constructs such as pain (e.g. pain intensity, duration, number of previous episodes), activity limitation (difficulty in sit to stand, walking, lifting, rolling over in bed), physical impairments (lumbar spine movement, neurological signs), participation (sick leave, type of work), psychological factors (expectations of recovery, fear of movement, self efficacy, catastrophisation, depression), and social factors (income, partner support, social isolation). In this hypothetical example, the results might indicate that there are three subgroups that best explain how people in the sample scored on these characteristics (these clustering techniques aim to identify the subgroup structure that minimises the within-subgroup variability and maximises the between-subgroup variability).

This approach is straightforward but as the number of clinical characteristics increases, interpretation and naming of the subgroup patterns of scoring can become more challenging. This is because with increasing complexity, the patterns may no longer be clinically recognisable. Moreover, the statistically optimal number of subgroups may become so high that it does not make practical sense.

### Novel approach of using statistical clustering techniques (low back pain example)

**Fig. 2:**
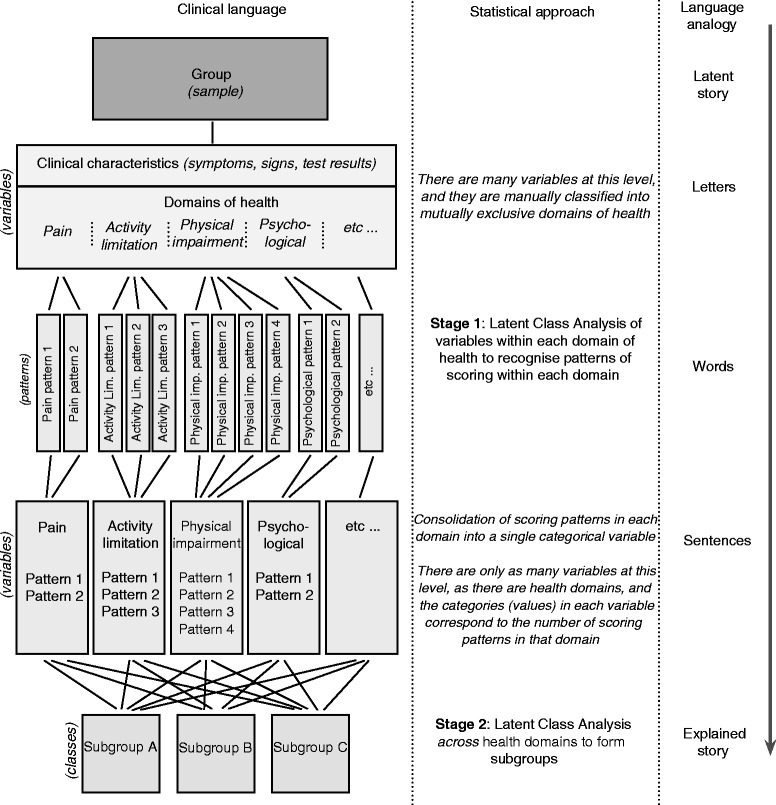
Novel approach to using statistical clustering techniques

The novel approach we suggest seeks to improve clinical interpretability and reduce sample size requirements by performing statistical clustering in two sequential stages. The first stage involves clustering using only clinical characteristics from *within* each health domain and therefore requires creating as many clustering models as there are health domains in the available data. This first stage produces scoring patterns within each domain. The second stage involves clustering using the scoring patterns from each health domain to identify subgroups *across* all the domains. Therefore, the scoring patterns identified in the first stage are treated as manifest categorical variables in the second stage. This principle is illustrated in Fig. 2.

### First stage clustering

Initially, the researcher needs to classify each available clinical characteristic as belonging exclusively to one health domain. The International Classification of Functioning, Disability and Health from the World Health Organisation is an example of a classification tool that can guide this process [20]. Some clinical characteristics may be ambiguous to classify and in such cases, classification decisions will necessarily be arbitrary.

Next, clustering is performed using only clinical characteristics from within each health domain. So extending our example, Latent Class Analysis could be initially performed using only the pain characteristics (pain intensity, duration and number of previous episodes). The results of the Latent Class Analysis would identify how many discrete patterns of scoring on the pain characteristics best explained the variance in the whole sample. For example, one scoring pattern might be labelled ‘high pain intensity/short duration’ if it mainly varied from the other patterns on these two pain characteristics. This process of Latent Class Analysis, interpretation and labelling of results would then be repeated for each health domain.

Then, new categorical variables would be formed, one for each domain, with the categories (values) in each variable corresponding to the number of scoring patterns in that domain. For example, a new pain variable might be formed containing three categories that are labelled ‘high pain intensity/short duration’, ‘high pain intensity/constant pain’, and ‘low pain intensity/long duration’.

One benefit of this first stage is potentially enhanced clinical interpretability of the results because the labelling only describes clinical characteristics from within one health domain and therefore each scoring pattern may be more recognisable. A second likely benefit is reduced sample size requirements because clustering within domains can be a powerful data-reduction technique. In our simple example, this first stage would have reduced the number of clinical characteristics (variables) being modelled from 16 (pain intensity, pain duration, pain behaviour, activity limitation, lumbar spine movement, neurological signs, sick leave, type of work, number of previous episodes, expectations of recovery, fear of movement, self efficacy, catastrophisation, depression, partner support and social isolation) to six (the domains of pain, activity limitation, participation, physical impairment, psychology, and social factors). That is because only the number of variables within each domain would be clustered during the first stage and only the number of variables that equals the number of domains would be clustered in the second stage. This data reduction would potentially be even greater in circumstances where some of the constructs are multi-dimensional and where greater numbers of clinical characteristics are being modelled.

### Second stage clustering

The second stage involves clustering, using the categorical variables derived from the first stage, to identify subgroups across all the domains. So, in our example, we would perform a second Latent Class Analysis modelling the six variables that each represents the scoring patterns within one health domain. It is likely that, relative to the traditional method for performing statistical clustering, the clinical interpretability of the results would be enhanced because the subgroups would be formed from fewer variables that already contain values (scoring patterns) that have clinically interpretable labels.

An analogy for this novel approach to statistical clustering is the use of written language to explain a story (Fig. 2). We start with an idea (a latent story) that we wish to meaningfully explain. We require letters (variables) to form words (scoring patterns) that can be assembled into sentences (derived variables for each health domain) to explain the story (subgroups). The traditional method for performing statistical clustering attempts to bridge from letters (variables) through to the explained story (subgroups) and is, at least theoretically, subsequently more difficult to read. However, it may also result in a more interesting and unexpected story.

In summary, the potential advantages of this novel approach are enhanced clinical interpretability of data-derived subgroups and reduced sample size requirements. Potential disadvantages of this method are: (i) that it is more time-consuming to build separate clustering models for each included health domain and perform statistical clustering in two stages, (ii) the first stage clustering may hide potentially important interactions between factors from different domains.

### Assessing clinical importance

**Fig. 3:**
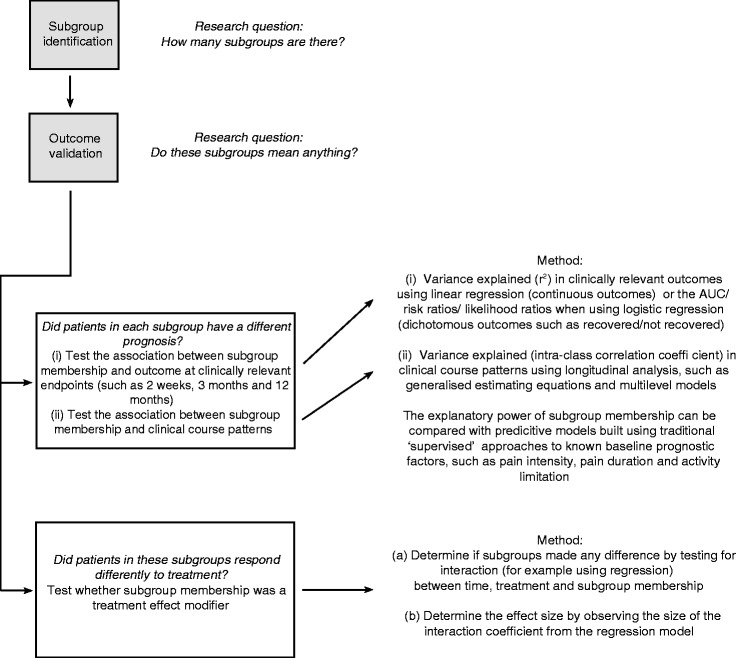
Determining the clinical relevance of subgroups identified using statistical clustering techniques

To determine if these subgroups are clinically important due to their having prognostic or treatment effect implications, they would need to be tested for their association with clinical outcomes and some methods for doing so are shown in Fig. 3. Those methods are divided into three lines of inquiry: (i) did patients in each subgroup have different prognoses?, (ii) did patients in these subgroups respond differently to treatment?, and (iii) how did the performance of this subgroup classification compare with other classification methods? This novel approach for applying statistical clustering techniques to identify clinically useful subgroups has potential benefits, but for any particular patient sample, the added value compared to the traditional approach needs to be evaluated. This can be tested by analysing the data using both approaches and comparing the interpretability of their results and the strength of their predictive capacity. For instance, either the traditional approach or the novel approach may explain more variance in clinically relevant outcomes and allow greater predictive accuracy when estimating the prognosis or treatment response of individual patients. So, all of the statistical approaches described in Fig. 3 can be used to also test whether one method for applying statistical clustering techniques is more useful in a given dataset. In the case of the chest pain data used as an example in the current study, we do not report whether the subgroups had prognostic or treatment effect implications, because the outcomes in the two data sources differed.

If statistically-derived subgroups show predictive validity at a clinically useful level, it can be helpful to then construct a clinical prediction rule that will allow clinicians to easily identify the subgroup membership of individual patients. This process requires identification of the most suitable set of clinical characteristics and creation of a scoring algorithm capable of allocating patients to the correct subgroup, while balancing operational simplicity with classification accuracy. There are a number of methods for doing this. Three examples of these methods are (i) the use of receiver operating characteristic (ROC) curve analysis and contingency tables [9], (ii) the use of logistic regression and scoring tables [8], and (iii) the use of more automated techniques such as Classification and Regression Tree Analysis [21].

Lastly, the type of clustering approaches described in this paper are hypothesis-setting, even when predictive accuracy has been demonstrated in an initial patient sample. There are subsequent stages of research needed to externally validate new subgrouping tools and adequately test their capacity to improve clinical effects and/or increase health system efficiency [9, 22].

## Results

### Example of this method applied (non-specific chest pain example)

Identical baseline variables from two clinical trials of non-specific chest pain in Denmark were combined to create test data on which to apply this method of analysis. The researchers working with these data had a suspicion that there may be two latent subgroups, one subgroup representing predominantly cardiogenic chest pain, and the other representing predominantly musculoskeletal chest pain.

One clinical trial was of two treatments for acute musculoskeletal chest pain undertaken in an emergency cardiology department and four chiropractic clinics, and the data from evaluating 305 prospective participants was included in the current study. Full details of the study design and recruitment procedures have been published previously [23, 24]. The other clinical trial was of manual therapy for chest pain in people diagnosed with or without cervico-thoracic angina, and the data from evaluating 275 prospective participants was included in the current study. Full details of that study’s design and recruitment procedures have also been published previously [25, 26]. Permission was obtained from the custodians (MJS and HWC) of each of these datasets for secondary use of the data within this project. Under Danish law, the secondary analysis of such de-identified data does not require separate ethics approval (The Act on Processing of Personal Data, December 2012, Section 5.2; Act on Research Ethics Review of Health Research Projects, October 2013, Section 14.2).

For both the single-stage and two-stage clustering, 69 variables were selected that traversed the health domains of demographics, previous history, psychological perception and coping, pain, activity limitation, diagnostic classification and musculoskeletal palpation findings, so as to illustrate data-derived subgroups that were identified using information across health domains. The number of variables differed across domains and also contained a variety of metrics. A detailed description of all variables is shown in Table 1. All variables contained 1 % or less missing data, except for the CCS angina classification variable (1.6 %), SF36 physical function sum score (3.5 %) and average chest pain episode duration (21.9 %). However, no data were imputed, as Latent Class Analysis copes with missing data.

**Table 1:** Description of variables

	Response options	Missing-ness
Demographics domain		
Age	Years of age	0 %
Gender	Female/male	0 %
Previous history domain		
Risk factors for cardiovascular disease sum score of hypercholesterolemia, hypertension, diabetes, family history of cardiac disease, previous or current smoking (all scored yes/no)	0 to 5	0 %
Psychological domain (patient perception and coping)		
Does the pain originate from the heart?	Yes/possibly/no	0.4 %
Does the pain originate from the muscles and joints?	Yes/possibly/no	0.2 %
Are you afraid?	Yes/no	0.2 %
Pain domain		
Average chest pain episode duration	<10 min/10 min-1 hour/> 1 hour/continuous	21.9 %
Pain description ‘crushing pain’	Yes/no	0 %
Pain description ‘tenderness’	Yes/no	0 %
Pain description ‘sharp pains’	Yes/no	0 %
Pain description ‘well defined’	Yes/no	0 %
Pain description ‘diffuse’	Yes/no	0 %
Pain description ‘burning’	Yes/no	0 %
Pain description ‘tingling’	Yes/no	0 %
Activity limitation domain		
SF36 physical function sum score	0 to 100 scale (high scores better)	3.5 %
Diagnostic classification domain		
Danish Cardiologist’s Society classification ‘angina’	No angina/typical/atypical/uncharacteristic/ unstable	0.2 %
Canadian Cardiovascular Society classification	Grades 0 to 3	1.6 %
Palpation domain		
Anterior		
Springing - tenderness sterno/xiphoid junction	Yes/no	<1 %
Springing - tenderness costosternal 2–3 right	Yes/no	<1 %
Springing - tenderness costosternal 2–3 left	Yes/no	<1 %
Springing - tenderness costosternal 3–4 right	Yes/no	<1 %
Springing - tenderness costosternal 3–4 left	Yes/no	<1 %
Springing - tenderness costosternal 4–5 right	Yes/no	<1 %
Springing - tenderness costosternal 4–5 left	Yes/no	<1 %
Springing - tenderness costosternal 5–6 right	Yes/no	<1 %
Springing - tenderness costosternal 5–6 left	Yes/no	<1 %
Springing - provoked pain sterno/xiphoid junction	Yes/no	<1 %
Springing - provoked pain costosternal 2–3 right	Yes/no	<1 %
Springing - provoked pain costosternal 2–3 left	Yes/no	<1 %
Springing - provoked pain costosternal 3–4 right	Yes/no	<1 %
Springing - provoked pain costosternal 3–4 left	Yes/no	<1 %
Springing - provoked pain costosternal 4–5 right	Yes/no	<1 %
Springing - provoked pain costosternal 4–5 left	Yes/no	<1 %
Springing - provoked pain costosternal 5–6 right	Yes/no	<1 %
Springing - provoked pain costosternal 5–6 left	Yes/no	<1 %
Muscle tenderness/pain pectoralis maj. left	No tenderness/tenderness/pain	<1 %
Muscle tenderness/pain pectoralis min. left	No tenderness/tenderness/pain	<1 %
Muscle tenderness/pain intercostalis 2–3 left	No tenderness/tenderness/pain	<1 %
Muscle tenderness/pain intercostalis 3–4 left	No tenderness/tenderness/pain	<1 %
Muscle tenderness/pain intercostalis 4–5 left	No tenderness/tenderness/pain	<1 %
Muscle tenderness/pain intercostalis 5–6 left	No tenderness/tenderness/pain	<1 %
Muscle tenderness/pain intercostalis 6–7 left	No tenderness/tenderness/pain	<1 %
Muscle tenderness/pain pectoralis maj. right	No tenderness/tenderness/pain	<1 %
Muscle tenderness/pain pectoralis min. right	No tenderness/tenderness/pain	<1 %
Muscle tenderness/pain intercostalis 2–3 right	No tenderness/tenderness/pain	<1 %
Muscle tenderness/pain intercostalis 3–4 right	No tenderness/tenderness/pain	<1 %
Muscle tenderness/pain intercostalis 4–5 right	No tenderness/tenderness/pain	<1 %
Muscle tenderness/pain intercostalis 5–6 right	No tenderness/tenderness/pain	<1 %
Muscle tenderness/pain intercostalis 6–7 right	No tenderness/tenderness/pain	<1 %
Posterior		
Tender c4-5 paraspinalpost either left or right	Yes/no	1 %
Tender c5-6 paraspinalpost either left or right	Yes/no	1 %
Tender c6-7 paraspinalpost either left or right	Yes/no	1 %
Tender c7-th1 paraspinalpost left or right	Yes/no	1 %
Tender th1-2 paraspinalpost left or right	Yes/no	1 %
Tender th2-3 paraspinalpost left or right	Yes/no	1 %
Tender th3-4 paraspinalpost left or right	Yes/no	1 %
Tender th4-5 paraspinalpost left or right	Yes/no	1 %
Tender th5-6 paraspinalpost left or right	Yes/no	1 %
Tender th6-7 paraspinalpost left or right	Yes/no	1 %
Tender th7-8 paraspinalpost left or right	Yes/no	1 %
Tender th8-9 paraspinalpost left or right	Yes/no	1 %
Vertebral springing th1-2	Yes/no	1 %
Vertebral springing th2-3	Yes/no	1 %
Vertebral springing th3-4	Yes/no	1 %
Vertebral springing th4-5	Yes/no	1 %
Vertebral springing th5-6	Yes/no	1 %
Vertebral springing th6-7	Yes/no	1 %
Vertebral springing th7-8	Yes/no	1 %
Vertebral springing th8-9	Yes/no	1 %

Latent Class Analysis was performed using Latent Gold (*version 4.5, Statistical Innovations, Belmont MA, USA*), and as use of a random seed start-point in the data can produce some slight variability in results, all analyses were performed five times. The appropriate number of clusters (subgroups) was chosen from the model with the lowest and most consistent Bayesian Information Criterion (BIC) across the five repetitions.

All subgroups and scoring patterns were independently named by two researchers (MJS and HWC) with content expertise and then a consensus was reached by discussion. The two experts had conducted the clinical trials and were very familiar with both the variables and the clinical context in which the trials occurred.

The results from the *single-stage clustering* are shown in Table 2. Five subgroups were identified which were subsequently named: (i) *Low activity limitation, high fear and few palpation findings (32 % of sample)*, (ii) *Typical and atypical angina, short episode, with pectoral tenderness (26 %)*, (iii) *no palpation findings (24 %)*, (iv) *Female, high fear, diffuse anterior tenderness (10 %)*, and (v) *Typical angina, short episode, high activity limitation, with pectoral tenderness (7 %)*.

**Table 2:** Results of the single-stage clustering of chest pain data

	Response option	Cluster1	Cluster2	Cluster3	Cluster4	Cluster5
Subgroup label		Low activity limitation, high fear, few palpation findings	Typical and atypical angina, short episode, pectoral tenderness	No palpation findings	Female, high fear, diffuse anterior tenderness	Typical angina, short episode, high activity limitation, pectoral tenderness
Cluster Size		32 %	26 %	24 %	10 %	7 %
Cluster membership probability,	Median (interquartile range)	100 % (100 % to 100 %)	100 % (100 % to 100 %)	100 % (100 % to 100 %)	100 % (100 % to 100 %)	100 % (100 % to 100 %)
Demographic domain						
Age	Mean (SD)	51.5 (11.0)	55.4 (9.4)	55.3 (10.3)	54.5 (10.2)	59.0 (9.0)
Gender	Female	42 %	33 %	27 %	93 %	36 %
Previous history domain						
Cardiovascular risk factor Index	Mean (SD)	1.9 (1.1)	2.1 (1.1)	2.0 (1.2)	2.1 (1.1)	2.4 (0.9)
Psychological domain						
Self-perceived ‘Pain from muscle or joints’	Possibly	43 %	42 %	36 %	41 %	43 %
	Yes	31 %	32 %	12 %	38 %	30 %
Self-perceived ‘Pain from heart’	Possibly	39 %	35 %	37 %	40 %	32 %
	Yes	22 %	52 %	47 %	29 %	59 %
Self-perceived ‘Afraid’	Yes	94 %	69 %	77 %	92 %	82 %
Pain domain						
Episode duration	<10 min	38 %	72 %	59 %	50 %	68 %
	10 min-1 hour	20 %	18 %	21 %	21 %	19 %
	>1 hour	9 %	4 %	6 %	7 %	4 %
	Continuous	34 %	7 %	14 %	22 %	9 %
Pain description ‘crushing pain’	Yes	73 %	92 %	80 %	82 %	92 %
Pain description ‘tenderness’	Yes	73 %	73 %	73 %	73 %	73 %
Pain description ‘sharp pains’	Yes	29 %	18 %	16 %	28 %	26 %
Pain description ‘well defined’	Yes	4 %	4 %	3 %	3 %	5 %
Pain description ‘diffuse’	Yes	4 %	10 %	3 %	2 %	8 %
Pain description ‘burning’	Yes	7 %	4 %	4 %	8 %	5 %
Pain description ‘tingling’	Yes	7 %	17 %	9 %	7 %	10 %
Activity limitation domain						
SF36 physical function sum-score	Mean (SD)	70.8 (9.9)	67.0 (9.4)	66.8 (11.9)	58.9 (16.2)	28.6 (8.9)
Diagnostic classification domain						
Danish Cardiologist’s Society classification ‘angina’	No angina	45 %	3 %	23 %	38 %	8 %
	Typical	9 %	52 %	31 %	27 %	77 %
	Atypical	17 %	32 %	24 %	15 %	6 %
	Uncharacteristic	30 %	14 %	21 %	20 %	10 %
	Unstable	0 %	0 %	1 %	0 %	0 %
Canadian Cardiovascular Society classification	Grade 0	65 %	12 %	27 %	35 %	3 %
	Grade 1	29 %	35 %	42 %	41 %	21 %
	Grade 2	6 %	48 %	30 %	23 %	63 %
	Grade 3	0 %	4 %	1 %	1 %	12 %
Palpation domain						
Sternoxiphoid springing tenderness	Yes	16 %	1 %	9 %	38 %	0 %
Costosternal springing tenderness 2/3 right	Yes	11 %	2 %	0 %	42 %	5 %
Costosternal springing tenderness 2/3 left	Yes	15 %	1 %	0 %	45 %	3 %
Costosternal springing tenderness 3/4 right	Yes	14 %	0 %	1 %	45 %	5 %
Costosternal springing tenderness 3/4 left	Yes	22 %	0 %	0 %	52 %	3 %
Costosternal springing tenderness 4/5 right	Yes	15 %	0 %	1 %	57 %	3 %
Costosternal springing tenderness 4/5 left	Yes	45 %	0 %	1 %	62 %	5 %
Costosternal springing tenderness 5/6 right	Yes	11 %	1 %	1 %	57 %	3 %
Costosternal springing tenderness 5/6 left	Yes	30 %	1 %	3 %	59 %	0 %
sternoxiphoid springing pain	Yes	3 %	0 %	3 %	7 %	0 %
Costosternal springing pain 2/3 right	Yes	2 %	1 %	0 %	7 %	2 %
Costosternal springing pain 2/3 left	Yes	3 %	1 %	0 %	8 %	0 %
Costosternal springing pain 3/4 right	Yes	3 %	0 %	1 %	9 %	2 %
Costosternal springing pain 3/4 left	Yes	8 %	0 %	0 %	8 %	0 %
Costosternal springing pain 4/5 right	Yes	3 %	0 %	0 %	14 %	2 %
Costosternal springing pain 4/5 left	Yes	19 %	0 %	0 %	12 %	0 %
Costosternal springing pain 5/6 right	Yes	3 %	0 %	0 %	10 %	0 %
Costosternal springing pain 5/6 left	Yes	16 %	0 %	0 %	12 %	0 %
Muscle tenderness pectoralis major left	Tenderness	26 %	72 %	8 %	47 %	61 %
	Pain	4 %	23 %	2 %	18 %	22 %
Muscle tenderness pectoralis minor left	Tenderness	34 %	82 %	4 %	61 %	77 %
	Pain	6 %	11 %	1 %	15 %	8 %
Muscle tenderness intercostal 2/3 left	Tenderness	15 %	50 %	1 %	69 %	55 %
	Pain	0 %	3 %	0 %	8 %	5 %
Muscle tenderness intercostal 3/4 left	Tenderness	20 %	30 %	0 %	80 %	25 %
	Pain	2 %	0 %	0 %	7 %	2 %
Muscle tenderness intercostal 4/5 left	Tenderness	26 %	13 %	1 %	86 %	13 %
	Pain	8 %	1 %	0 %	10 %	2 %
Muscle tenderness intercostal 5/6 left	Tenderness	33 %	5 %	2 %	78 %	5 %
	Pain	9 %	0 %	0 %	15 %	0 %
Muscle tenderness intercostal 6/7 left	Tenderness	22 %	1 %	1 %	75 %	3 %
	Pain	7 %	0 %	0 %	13 %	0 %
Muscle tenderness pectoralis major right	Tenderness	20 %	71 %	9 %	49 %	73 %
	Pain	2 %	22 %	0 %	18 %	13 %
Muscle tenderness pectoralis minor right	Tenderness	23 %	76 %	3 %	60 %	73 %
	Pain	1 %	9 %	0 %	15 %	12 %
Muscle tenderness intercostal 2/3 right	Tenderness	13 %	48 %	0 %	76 %	54 %
	Pain	1 %	0 %	0 %	7 %	2 %
Muscle tenderness intercostal 3/4 right	Tenderness	10 %	25 %	1 %	81 %	25 %
	Pain	1 %	0 %	0 %	5 %	2 %
Muscle tenderness intercostal 4/5 right	Tenderness	12 %	9 %	0 %	88 %	5 %
	Pain	0 %	0 %	0 %	7 %	0 %
Muscle tenderness intercostal 5/6 right	Tenderness	14 %	4 %	0 %	82 %	0 %
	Pain	0 %	0 %	0 %	10 %	0 %
Muscle tenderness intercostal 6/7 right	Tenderness	8 %	2 %	0 %	79 %	0 %
	Pain	1 %	0 %	0 %	10 %	0 %
Tenderness c4/5 paraspinal posterior	Yes	10 %	5 %	2 %	27 %	3 %
Tenderness c5/6 paraspinal posterior	Yes	16 %	8 %	4 %	26 %	3 %
Tenderness c6/7 paraspinal posterior	Yes	14 %	5 %	3 %	17 %	3 %
Tenderness c7/th1 paraspinal posterior	Yes	7 %	11 %	1 %	18 %	10 %
Tenderness th1/2 paraspinal posterior	Yes	14 %	27 %	3 %	29 %	38 %
Tenderness th2/3 paraspinal posterior	Yes	29 %	50 %	5 %	50 %	64 %
Tenderness th3/4 paraspinal posterior	Yes	37 %	46 %	3 %	58 %	47 %
Tenderness th4/5 paraspinal posterior	Yes	45 %	24 %	3 %	56 %	29 %
Tenderness th5/6 paraspinal posterior	Yes	46 %	9 %	2 %	45 %	13 %
Tenderness th6/7 paraspinal posterior	Yes	42 %	2 %	1 %	40 %	5 %
Tenderness th7/8 paraspinal posterior	Yes	27 %	1 %	1 %	36 %	3 %
Tenderness th8/9 paraspinal posterior	Yes	16 %	1 %	0 %	22 %	3 %
Posterior vertebral springing th1/2	Yes	7 %	4 %	0 %	5 %	12 %
Posterior vertebral springing th2/3	Yes	12 %	29 %	1 %	14 %	25 %
Posterior vertebral springing th3/4	Yes	18 %	37 %	0 %	31 %	25 %
Posterior vertebral springing th4/5	Yes	15 %	19 %	0 %	31 %	20 %
Posterior vertebral springing th5/6	Yes	19 %	6 %	0 %	16 %	10 %
Posterior vertebral springing th6/7	Yes	18 %	1 %	1 %	16 %	2 %
Posterior vertebral springing th7/8	Yes	14 %	1 %	1 %	14 %	0 %
Posterior vertebral springing th8/9	Yes	12 %	0 %	2 %	19 %	0 %

The results from the first stage of the *two-stage clustering* are shown in Table 3, including the names of the two to six scoring patterns that were identified within each health domain. The results from the second stage are shown in Tables 4 and 5, which identified two subgroups which were subsequently named: (i) *Uncertain of cause and fearful, but not heart-related - with local thoracic 5/6 palpation findings* (*51 % of sample*), and (ii) *Believes pain to be heart- or musculoskeletal-related with crushing pain, local thoracic 2/3 signs, and pectoral tenderness* (*49 %*)*.* In Table 4, these two subgroups are described using the proportions of individuals in each of the first stage subgroups. In Table 5, these two subgroups are described in the measurement units of the original variables.

**Table 3:** Results of the first stage of two-stage clustering of chest pain data

Response option							
Domain: Demographic							
**2 clusters**		Cluster1	Cluster2				
***Scoring pattern label***		*Older*	*Younger*				
Cluster Size		51 %	49 %				
Cluster membership probability	Median (interquartile range)	90 % (79 % to 96 %)	93 % (78 % to 100 %)				
Age (years)	Mean (SD)	62.6 (5.2)	45.3 (6.3)				
Gender	Female	43 %	39 %				
**Domain: Previous history**							
**1 cluster**		*Previous history did not discriminate subgroups in two-stage clustering*					
**Domain: Psychological**							
**5 clusters**		Cluster1	Cluster2	Cluster3	Cluster4	Cluster5	
***Scoring pattern label***		*Uncertain of cause of pain and fearful*	*Believes cause from heart*	*Believes both from heart and musculoskeletal*	*Believes cause from musculoskeletal and less fearful*	*Believes cause from other cause and less fearful*	
Cluster Size		44 %	23 %	16 %	11 %	6 %	
Cluster membership probability	Median (interquartile range)	100 % (92 % to 100 %)	99. % (99 % to 100 %)	100 % (100 % to 100 %)	95 % (95 % to 96 %)	77 % (77 % to 100 %)	
Self-perceived ‘Pain from muscle or joints’	No	10 %	100 %	0 %	0 %	75 %	
	Possibly	85 %	0 %	1 %	13 %	25 %	
	Yes	5 %	0 %	99 %	87 %	0 %	
Self-perceived ‘Pain from heart’	No	15 %	0 %	0 %	99 %	94 %	
	Possibly	83 %	1 %	1 %	1 %	6 %	
	Yes	3 %	99 %	99 %	0 %	0 %	
Self-perceived ‘Afraid’	Yes	99 %	71 %	83 %	56 %	50 %	
**Domain: Pain**							
**2 clusters**		Cluster1	Cluster2				
***Scoring pattern label***		*Crushing pain*	*Not crushing pain*				
Cluster Size		77 %	23 %				
Cluster membership probability, median (interquartile range)		97 % (94 % to 97 %)	99 % (96 % to 99 %)				
Episode duration	< 10 min	59 %	50 %				
	10 min-1 hour	20 %	20 %				
	> 1 hour	5 %	7 %				
	Continuous	16 %	24 %				
Pain description ‘crushing pain’	Yes	100 %	25 %				
Pain description ‘tenderness’	Yes	3 %	3 %				
Pain description ‘sharp pains’	Yes	14 %	52 %				
Pain description ‘well defined’	Yes	3 %	6 %				
Pain description ‘diffuse’	Yes	6 %	5 %				
Pain description ‘burning’	Yes	4 %	8 %				
Pain description ‘tingling’	Yes	8 %	19 %				
**Domain: Activity limitation**							
**3 clusters**		Cluster1	Cluster2	Cluster3			
*Scoring pattern label*		*None or light activity limitation*	*Moderate activity limitation*	*Severe activity limitation*			
Cluster Size		82 %	11 %	8 %			
Cluster membership probability, median (interquartile range)		100 % (100 % to 100 %)	86 % (86 % to 86 %)	99 % (82 % to 100 %)			
SF36 physical function sum-score	Mean (SD)	71.2 (7.4)	49.0 (4.6)	27.1 (6.9)			
**Domain: Diagnostic classification**							
**3 clusters**		Cluster1	Cluster2	Cluster3			
***Scoring pattern label***		*Not heart*	*Typical angina*	*Maybe angina*			
Cluster Size		37 %	35 %	28 %			
Cluster membership probability	Median (interquartile range)	100 % (94 % to 100 %)	100 % (85 % to 100 %)	97 % (81 % to 100 %)			
Danish Cardiologist’s Society	No angina	64 %	3 %	2 %			
classification ‘angina’	Typical	0 %	89 %	4 %			
	Atypical	1 %	8 %	65 %			
	Uncharacteri-stic	34 %	0 %	29 %			
	Unstable	0 %	1 %	0 %			
Canadian Cardiovascular Society	Grade 0	87 %	0 %	8 %			
classification	Grade 1	13 %	17 %	84 %			
	Grade 2	0 %	76 %	8 %			
	Grade 3	0 %	7 %	0 %			
**Domain: Palpation**							
**6 clusters**		Cluster1	Cluster2	Cluster3	Cluster4	Cluster5	Cluster6
***Scoring pattern label***		*No palpation findings*	*Local 5/6 signs and intercostal tenderness*	*Local 2/3/4 signs and pectoral tenderness*	*Pectoral tenderness*	*Paraspinal and pectoral tenderness*	*Generalised tenderness*
Cluster Size		22 %	19 %	18 %	16 %	16 %	8 %
Cluster membership probability, median (interquartile range)		100 % (100 % to 100 %)	100 % (100 % to 100 %)	100 % (100 % to 100 %)	100 % (100 % to 100 %)	100 % (100 % to 100 %)	100 % (100 % to 100 %)
Sternoxiphoid springing tenderness	Yes	10 %	22 %	1 %	1 %	10 %	34 %
Costosternal tenderness 2/3 right	Yes	0 %	5 %	1 %	4 %	16 %	52 %
Costosternal tenderness 2/3 left	Yes	1 %	7 %	0 %	2 %	19 %	56 %
Costosternal tenderness 3/4 right	Yes	2 %	13 %	1 %	3 %	12 %	52 %
Costosternal tenderness 3/4 left	Yes	2 %	25 %	0 %	0 %	18 %	54 %
Costosternal tenderness 4/5 right	Yes	1 %	28 %	1 %	1 %	4 %	51 %
Costosternal tenderness 4/5 left	Yes	2 %	77 %	1 %	1 %	10 %	52 %
Costosternal tenderness 5/6 right	Yes	1 %	27 %	1 %	1 %	0 %	51 %
Costosternal tenderness 5/6 left	Yes	3 %	55 %	0 %	0 %	6 %	51 %
sternoxiphoid springing pain	Yes	4 %	3 %	0 %	0 %	4 %	4 %
Costosternal springing pain 2/3 right	Yes	0 %	0 %	0 %	4 %	1 %	8 %
Costosternal springing pain 2/3 left	Yes	0 %	1 %	0 %	1 %	4 %	10 %
Costosternal springing pain 3/4 right	Yes	0 %	2 %	1 %	2 %	2 %	10 %
Costosternal springing pain 3/4 left	Yes	1 %	7 %	0 %	0 %	5 %	10 %
Costosternal springing pain 4/5 right	Yes	0 %	8 %	1 %	0 %	1 %	6 %
Costosternal springing pain 4/5 left	Yes	1 %	36 %	0 %	0 %	0 %	4 %
Costosternal springing pain 5/6 right	Yes	0 %	9 %	0 %	0 %	0 %	4 %
Costosternal springing pain 5/6 left	Yes	0 %	29 %	0 %	1 %	2 %	6 %
Muscle tenderness pectoralis major left	Tenderness	1 %	19 %	78 %	62 %	38 %	54 %
	Pain	0 %	3 %	20 %	28 %	4 %	24 %
Muscle tenderness pectoralis minor left	Tenderness	2 %	31 %	83 %	75 %	40 %	66 %
	Pain	0 %	6 %	13 %	8 %	4 %	20 %
Muscle tenderness intercostal 2/3 left	Tenderness	1 %	6 %	58 %	42 %	24 %	84 %
	Pain	1 %	0 %	3 %	1 %	0 %	12 %
Muscle tenderness intercostal 3/4 left	Tenderness	1 %	20 %	36 %	20 %	18 %	90 %
	Pain	1 %	2 %	0 %	0 %	1 %	8 %
Muscle tenderness intercostal 4/5 left	Tenderness	3 %	46 %	12 %	10 %	5 %	91 %
	Pain	1 %	15 %	0 %	1 %	1 %	6 %
Muscle tenderness intercostal 5/6 left	Tenderness	2 %	57 %	5 %	3 %	4 %	89 %
	Pain	0 %	21 %	0 %	0 %	0 %	6 %
Muscle tenderness intercostal 6/7 left	Tenderness	1 %	42 %	1 %	1 %	0 %	82 %
	Pain	0 %	14 %	0 %	1 %	1 %	8 %
Muscle tenderness pectoralis major right	Tenderness	0 %	16 %	75 %	74 %	28 %	56 %
	Pain	0 %	0 %	21 %	16 %	4 %	24 %
Muscle tenderness pectoralis minor right	Tenderness	1 %	17 %	80 %	68 %	33 %	66 %
	Pain	0 %	0 %	11 %	7 %	1 %	20 %
Muscle tenderness intercostal 2/3 right	Tenderness	0 %	9 %	59 %	38 %	16 %	90 %
	Pain	0 %	1 %	0 %	1 %	1 %	8 %
Muscle tenderness intercostal 3/4 right	Tenderness	1 %	15 %	32 %	15 %	9 %	90 %
	Pain	0 %	0 %	0 %	1 %	1 %	6 %
Muscle tenderness intercostal 4/5 right	Tenderness	0 %	27 %	10 %	4 %	4 %	90 %
	Pain	0 %	2 %	0 %	0 %	0 %	4 %
Muscle tenderness intercostal 5/6 right	Tenderness	0 %	30 %	3 %	1 %	0 %	89 %
	Pain	0 %	4 %	0 %	0 %	0 %	4 %
Muscle tenderness intercostal 6/7 right	Tenderness	0 %	17 %	2 %	2 %	0 %	84 %
	Pain	0 %	5 %	0 %	0 %	0 %	4 %
Tenderness c4/5 paraspinal posterior	Yes	3 %	6 %	6 %	1 %	17 %	27 %
Tenderness c5/6 paraspinal posterior	Yes	4 %	13 %	11 %	3 %	20 %	26 %
Tenderness c6/7 paraspinal posterior	Yes	3 %	13 %	8 %	3 %	12 %	19 %
Tenderness c7/th1 paraspinal posterior	Yes	1 %	8 %	18 %	0 %	9 %	21 %
Tenderness th1/2 paraspinal posterior	Yes	2 %	14 %	50 %	0 %	20 %	31 %
Tenderness th2/3 paraspinal posterior	Yes	3 %	27 %	89 %	0 %	46 %	51 %
Tenderness th3/4 paraspinal posterior	Yes	1 %	33 %	78 %	0 %	54 %	61 %
Tenderness th4/5 paraspinal posterior	Yes	1 %	38 %	40 %	1 %	59 %	59 %
Tenderness th5/6 paraspinal posterior	Yes	1 %	45 %	13 %	0 %	53 %	41 %
Tenderness th6/7 paraspinal posterior	Yes	1 %	43 %	1 %	0 %	45 %	35 %
Tenderness th7/8 paraspinal posterior	Yes	3 %	27 %	2 %	0 %	26 %	33 %
Tenderness th8/9 paraspinal posterior	Yes	2 %	12 %	0 %	0 %	17 %	25 %
Posterior vertebral springing th1/2	Yes	0 %	8 %	10 %	2 %	4 %	2 %
Posterior vertebral springing th2/3	Yes	1 %	7 %	41 %	11 %	18 %	17 %
Posterior vertebral springing th3/4	Yes	0 %	21 %	51 %	11 %	21 %	26 %
Posterior vertebral springing th4/5	Yes	0 %	15 %	31 %	5 %	18 %	28 %
Posterior vertebral springing th5/6	Yes	0 %	14 %	12 %	0 %	24 %	17 %
Posterior vertebral springing th6/7	Yes	1 %	19 %	2 %	0 %	19 %	11 %
Posterior vertebral springing th7/8	Yes	2 %	12 %	1 %	2 %	14 %	11 %
Posterior vertebral springing th8/9	Yes	5 %	9 %	1 %	0 %	10 %	19 %

**Table 4:** Results of the second stage of two-stage clustering (described using the prevalence of first stage cluster membership)

Domain		Cluster 1	Cluster 2
Subgroup label		Uncertain of cause and fearful, but not heart-related - with local thoracic 5/6 palpation findings.	Believes pain to be heart- or musculoskelet-al-related with crushing pain, and local thoracic 2/3 signs and pectoral tenderness.
Cluster size		51 %	49 %
Cluster membership probability	Median (interquartile range)	100 % (99 % to 100 %)	100 % (98 % to 100 %)
Demographic	Younger	42 %*	62 %
	Older	58 %	38 %
Previous history		0 %	0 %
	Only one cluster	100 %	100 %
Psychological	Uncertain of cause of pain and fearful	88 %	2 %
	Believes cause from heart	1 %	44 %
	Believes both from heart and musculoskeletal	0 %	31 %
	Believes cause from musculoskeletal and less fearful	6 %	14 %
	Believes cause from other cause and less fearful	5 %	8 %
Pain	Crushing pain	74 %	89 %
	Not crushing pain	26 %	11 %
Activity limitation	None or light activity limitation	88 %	76 %
	Moderate activity limitation	8 %	13 %
	Severe activity limitation	4 %	11 %
Diagnostic classification	Not heart	71 %	1 %
	Typical angina	13 %	61 %
	Maybe angina	16 %	38 %
Palpation	No palpation findings	23 %	23 %
	Local 5/6 signs and intercostal tenderness	36 %	1 %
	Local 2/3/4 signs and pectoral tenderness	4 %	32 %
	Pectoral tenderness	3 %	30 %
	Paraspinal and pectoral tenderness	23 %	8 %
	Generalised tenderness	11 %	6 %

*Proportions are those of the people within each cluster on each domain (vertical proportions), as each category within a domain is mutually exclusive

**Table 5:** Results of the second stage of two-stage clustering (described in the measurement units of the original variables)

	Response option	Cluster1	Cluster2
Subgroup label		Uncertain of cause and fearful, but not heart-related - with local thoracic 5/6 palpation findings.	Believes pain to be heart- or musculoskelet-al-related with crushing pain, and local thoracic 2/3 signs and pectoral tenderness.
Demographic domain			
Age	Mean (SD)	52.2 (11.0)	56.5 (9.2)
Gender	Female	44 %	39 %
Previous history domain			
Cardiovascular risk factor Index	Mean (SD)	1.9 (1.1)	22.2 (1.1)
Psychological domain			
Self-perceived ‘Pain from muscle or joints’	Possibly	79 %	1 %
	Yes	9 %	46 %
Self-perceived ‘Pain from heart’	Possibly	73 %	1 %
	Yes	3 %	76 %
Self-perceived ‘Afraid’	Yes	94 %	71 %
Pain domain			
Episode duration	<10 min	34 %	57 %
	10 min-1 hour	24 %	20 %
	>1 hour	5 %	6 %
	Continuous	37 %	17 %
Pain description ‘crushing pain’	Yes	76 %	90 %
Pain description ‘tenderness’	Yes	0 %	6 %
Pain description ‘sharp pains’	Yes	25 %	20 %
Pain description ‘well defined’	Yes	3 %	4 %
Pain description ‘diffuse’	Yes	2 %	8 %
Pain description ‘burning’	Yes	6 %	5 %
Pain description ‘tingling’	Yes	7 %	13 %
Activity limitation domain			
SF36 physical function sum-score	Mean (SD)	67.8 (SD 13.4)	61.5 (SD 16.1)
Diagnostic classification domain			
Danish Cardiologist’s Society	No angina	49 %	0 %
classification ‘angina’	Typical	9 %	56 %
	Atypical	14 %	30 %
	Uncharacteristic	28 %	14 %
	Unstable	0 %	0 %
Canadian Cardiovascular Society	Grade 0	67 %	1 %
classification	Grade 1	21 %	47 %
	Grade 2	11 %	46 %
	Grade 3	0 %	5 %
Palpation domain			
Sternoxiphoid springing tend	Yes	21 %	2 %
Costosternal springing tenderness 2/3 right	Yes	13 %	4 %
Costosternal springing tenderness 2/3 left	Yes	15 %	4 %
Costosternal springing tenderness 3/4 right	Yes	17 %	3 %
Costosternal springing tenderness 3/4 left	Yes	23 %	3 %
Costosternal springing tenderness 4/5 right	Yes	20 %	1 %
Costosternal springing tenderness 4/5 left	Yes	40 %	2 %
Costosternal springing tenderness 5/6 right	Yes	18 %	1 %
Costosternal springing tenderness 5/6 left	Yes	32 %	1 %
sternoxiphoid springing pain	Yes	5 %	0 %
Costosternal springing pain 2/3 right	Yes	2 %	1 %
Costosternal springing pain 2/3 left	Yes	3 %	1 %
Costosternal springing pain 3/4 right	Yes	3 %	1 %
Costosternal springing pain 3/4 left	Yes	6 %	1 %
Costosternal springing pain 4/5 right	Yes	5 %	0 %
Costosternal springing pain 4/5 left	Yes	15 %	0 %
Costosternal springing pain 5/6 right	Yes	4 %	0 %
Costosternal springing pain 5/6 left	Yes	13 %	0 %
Muscle tenderness pectoralis major left	Tenderness	23 %	54 %
Costosternal springing pain 5/6 left	Pain	6 %	17 %
Muscle tenderness pectoralis minor left	Tenderness	31 %	60 %
	Pain	7 %	7 %
Muscle tenderness intercostal 2/3 left	Tenderness	18 %	41 %
	Pain	1 %	3 %
Muscle tenderness intercostal 3/4 left	Tenderness	23 %	25 %
	Pain	2 %	1 %
Muscle tenderness intercostal 4/5 left	Tenderness	29 %	14 %
	Pain	7 %	1 %
Muscle tenderness intercostal 5/6 left	Tenderness	32 %	9 %
	Pain	9 %	0 %
Muscle tenderness intercostal 6/7 left	Tenderness	21 %	7 %
	Pain	7 %	0 %
Muscle tenderness pectoralis major right	Tenderness	21 %	55 %
	Pain	3 %	15 %
Muscle tenderness pectoralis minor right	Tenderness	25 %	54 %
	Pain	2 %	8 %
Muscle tenderness intercostal 2/3 right	Tenderness	18 %	39 %
	Pain	2 %	1 %
Muscle tenderness intercostal 3/4 right	Tenderness	17 %	23 %
	Pain	1 %	0 %
Muscle tenderness intercostal 4/5 right	Tenderness	20 %	11 %
	Pain	1 %	0 %
Muscle tenderness intercostal 5/6 right	Tenderness	21 %	7 %
	Pain	2 %	0 %
Muscle tenderness intercostal 6/7 right	Tenderness	16 %	6 %
	Pain	2 %	0 %
Tenderness c4/5 paraspinal posterior	Yes	11 %	5 %
Tenderness c5/6 paraspinal posterior	Yes	16 %	6 %
Tenderness c6/7 paraspinal posterior	Yes	12 %	5 %
Tenderness c7/th1 paraspinal posterior	Yes	7 %	10 %
Tenderness th1/2 paraspinal posterior	Yes	14 %	22 %
Tenderness th2/3 paraspinal posterior	Yes	27 %	40 %
Tenderness th3/4 paraspinal posterior	Yes	32 %	36 %
Tenderness th4/5 paraspinal posterior	Yes	35 %	23 %
Tenderness th5/6 paraspinal posterior	Yes	35 %	11 %
Tenderness th6/7 paraspinal posterior	Yes	31 %	6 %
Tenderness th7/8 paraspinal posterior	Yes	22 %	4 %
Tenderness th8/9 paraspinal posterior	Yes	16 %	1 %
Posterior vertebral springing th1/2	Yes	6 %	4 %
Posterior vertebral springing th2/3	Yes	9 %	21 %
Posterior vertebral springing th3/4	Yes	16 %	25 %
Posterior vertebral springing th4/5	Yes	14 %	15 %
Posterior vertebral springing th5/6	Yes	14 %	7 %
Posterior vertebral springing th6/7	Yes	14 %	3 %
Posterior vertebral springing th7/8	Yes	10 %	3 %
Posterior vertebral springing th8/9	Yes	11 %	1 %

A cross-tabulation of the cluster membership between the five subgroups from single-stage clustering and the two subgroups from two-stage clustering is shown in Table 6. One of the subgroups from the single-stage clustering was split across the two subgroups from two-stage clustering, but the rest of the single-stage subgroups were largely represented in only one of the two-stage subgroups.

**Table 6:** Cross-tabulation of cluster membership

	Two-stage clustering	
	Subgroup 1	Subgroup 2
	Uncertain of cause and fearful, but not heart-related - with local thoracic 5/6 palpation findings.	Believes pain to be heart- or musculoskeletal-related with crushing pain, and local thoracic 2/3 signs and pectoral tenderness.
Single stage clustering		
Subgroup 1		
Low activity limitation, high fear, few palpation findings	173	13
Subgroup 2		
Typical and atypical angina, short episode, pectoral tenderness	9	144
Subgroup 3		
No palpation findings	64	
Subgroup 4		
Female, high fear, diffuse anterior tenderness	44	16
Subgroup 5		
Typical angina, short episode, high activity limitation, pectoral tenderness	5	35

## Discussion

We have introduced a novel approach to using statistical clustering techniques to identify clinically useful subgroups of patients. It has been described using the hypothetical example of low back pain and then illustrated using an applied example of chest pain data. From those cross-sectional chest pain data, we reported the baseline subgroups detected using the traditional approach and the baseline subgroups detected using the novel approach.

We anticipated that two-stage clustering might result in more clinically interpretable subgrouping than traditional one-stage clustering, due to its giving equal potential weight to variables from each domain. In this chest pain sample, the two approaches resulted in two and five subgroups respectively, both of which appeared clinically interpretable and reasonably recognisable. The two subgroups described by the two-stage approach approximated classic textbook descriptions of the common diagnostic criteria for musculoskeletal chest pain (cluster 1) and atypical angina chest pain (cluster 2), and suggest that our suspicion that these data contained two subgroups may have been correct. An advantage of this approach was that two fairly distinct groups emerged that could be conceptualised as representing two different conditions. In addition, these two data-driven subgroups also differed quite markedly on patient beliefs about the anatomical cause of their condition. Such an additional finding could motivate a new view on these classic descriptions.

In comparison, the five subgroups described by the single-stage approach displayed less distinct differences. However, our overall subjective impression was that these five subgroups might also be clinically recognisable, as non-specific chest pain is a very complex complaint caused by a range of life-threatening and non-life threatening conditions. A high level of complexity in symptom presentation is in good concordance with our clinical experience that chest pain episodes are often multi-dimensional experiences accompanied by strong emotional response, that are not very well described by only the commonly defined textbook categories.

The cross-tabulation of the cluster membership between the five subgroups from single-stage clustering and the two subgroups from two-stage clustering showed evidence that both approaches seemed to reflect a similar latent data structure. However, as the single-stage clustering approach models the latent data structure of the whole data and the two-stage approach initially models the latent data structure within each domain, differences in subgroup membership across these approaches are to be expected. It should be recognised that the two-stage approach is not intended to find the identical cluster structure, but is a way to explore an alternative cluster structure. It is possible that the novel approach that we have presented may, in some circumstances, not closely mimic the data structure identified with single-stage clustering, but the results may nonetheless be more clinically useful. So it is important that clinical researchers who use two-stage clustering should judge, on a dataset by dataset basis, whether single or two-stage clustering provides more clinically interpretable subgroups that have better face validity and predictive validity.

Two-stage clustering is a form of variable reduction, as the second stage clustering involves fewer variables than the first stage. There are other methods available for variable reduction, such as Principal Components Analysis and Factor Analysis [27]. Those methods seek uni-dimensionality by identifying variables of the whole dataset that are highly correlated. However, many health domains are multi-dimensional and there may be clinical merit in retaining that knowledge. Therefore, LCA within health domains is not seeking uni-dimensionality but is identifying different within-domain scoring patterns and preserving those patterns with a new synthetic domain variable. Similarly, Principal Components Analysis and Factor Analysis are ‘variable-centered’ in that they seek to identify highly correlated variables, whereas LCA is ‘patient-centered’ in that it seeks to identify people whose scoring patterns are similar. Variable reduction can also be based on content, where content experts select the variables that are the most representative for each health domain and only use those selected variables in single-stage clustering. However, clustering approaches are often applied when there is inadequate prior knowledge about which measurements would best inform subgroup formation.

## Conclusions

In conclusion, we have suggested a new approach to using statistical clustering techniques to identify clinically useful subgroups of patients. Research designs, statistical methods and outcome metrics suitable for performing that testing have also been described. While the paper illustrates this approach using a practical example of (chest pain) baseline data, due to limitations in the data, it does not extend that analysis into testing the example’s clinical importance or implications for prognosis and treatment. We are undertaking such analysis on data from primary and secondary care patients with low back pain, and the results will be comprehensively reported in subsequent papers. This novel statistical approach has a number of potential benefits but requires broad testing, in multiple patient samples, to determine if it is useful. That is likely to be context-specific, depending on the characteristics of the available data and the research question being asked of it.
